# Lidocaine Versus Amiodarone in Patients With Shockable Out-of-Hospital Cardiac Arrest: A Target Trial Emulation

**DOI:** 10.1097/CCM.0000000000007265

**Published:** 2026-07-15

**Authors:** Georg Gelbenegger, Sheldon Cheskes, Ian R. Drennan, Steve Lin, Bernd Jilma, Markus Zeitlinger, Damon C. Scales, Anselm Jorda

**Affiliations:** 1 Department of Clinical Pharmacology, Medical University of Vienna, Vienna, Austria.; 2 Institute of Medical Sciences, University of Toronto, Toronto, ON, Canada.; 3 Sunnybrook Centre for Prehospital Medicine, Toronto, ON, Canada.; 4 Sunnybrook Research Institute and Department of Emergency Services, Sunnybrook Health Science Centre, Toronto, ON, Canada.; 5 Department of Family and Community Medicine, Division of Emergency Medicine, University of Toronto, Toronto, ON, Canada.; 6 Li Ka Shing Knowledge Institute, St. Michaels Hospital, Toronto, ON, Canada.; 7 Institute of Health Policy, Management and Evaluation, Dalla Lana School of Public Health, University of Toronto, Toronto, ON, Canada.; 8 Department of Critical Care Medicine, Sunnybrook Health Sciences Centre, Toronto, ON, Canada.

**Keywords:** advanced life support, antiarrhythmic, cardiopulmonary resuscitation, defibrillation, ventricular fibrillation

## Abstract

**OBJECTIVES:**

Amiodarone or lidocaine are recommended for the treatment of shockable out-of-hospital cardiac arrest (OHCA). Previous comparisons between the two antiarrhythmic drugs were inconclusive.

**DESIGN:**

This analysis included data from the Resuscitation Outcomes Consortium Cardiac Epidemiologic Registry 3 (2011–2015). A target trial framework was used to overcome confounding inherent in observational comparisons.

**SETTING:**

Data from patients with OHCA were prospectively collected by emergency medical services (EMS) at multiple North American sites.

**Patients:**

Adults with nontraumatic OHCA and an initial shockable rhythm who received at least three defibrillation attempts were included. Time zero was defined as first antiarrhythmic drug administration, at which eligibility criteria had to be met.

**INTERVENTIONS:**

Two antiarrhythmic treatment strategies were compared: lidocaine vs. amiodarone.

**MEASUREMENTS AND MAIN RESULTS:**

The primary outcome was survival to hospital discharge. The key secondary outcome was favorable neurologic outcome at discharge (modified Rankin scale score of 3 or less). Group differences were adjusted using inverse probability weighting (key covariates: age, sex, location of OHCA, witness status, bystander resuscitation, automated external defibrillator shock, elapsed time from call to advanced life support arrival/first EMS shock/advanced airway, intraosseous access) and a multiple logistic regression model. Of 2451 patients included, 987 received lidocaine and 1464 received amiodarone. The adjusted percentage point difference in survival to hospital discharge was 2.8% favoring lidocaine (95% CI, –0.6 to 6.2); the estimated survival probabilities were 26.2% (275/987) with lidocaine and 23.5% (329/1464) with amiodarone. No difference was observed in the estimated probability of favorable neurologic outcome at discharge between the lidocaine group (17.7%) and the amiodarone group (16.2%) (adjusted difference, 1.5%; 95% CI, –1.5 to 4.5).

**CONCLUSIONS:**

No statistically significant difference in survival or favorable neurologic outcome was observed between lidocaine and amiodarone; however, the wide CIs cannot exclude a clinically meaningful benefit for lidocaine. These findings are consistent with randomized controlled trials, but residual confounding cannot be excluded.

**KEY POINTS**

**Question**: Is the use of lidocaine or amiodarone associated with higher rates of survival to hospital discharge in shockable out-of-hospital cardiac arrest?

**Findings**: In this target trial emulation, the estimated probability of survival to hospital discharge was 26.2% in the lidocaine group and 23.5% in the amiodarone group (adjusted percentage point difference, 2.8%; 95% CI, –0.6 to 6.2).

**Meaning**: No statistically significant difference in survival was observed between lidocaine and amiodarone; however, the wide CI cannot exclude a clinically meaningful benefit for lidocaine.

Out-of-hospital cardiac arrest (OHCA) is a significant cause of morbidity and mortality worldwide, with an estimated survival of less than 10% (1, 2). Although patients presenting with ventricular fibrillation (VF) or pulseless ventricular tachycardia (pVT) are considered to have a more favorable prognosis because of their potential responsiveness to defibrillation (3), a subset of patients remain in VF despite multiple defibrillation attempts (4), termed refractory VF (inclusion criterion for the present study, with antiarrhythmic therapy typically administered after the third shock). In patients with refractory VF, amiodarone and lidocaine are widely used to promote successful defibrillation and prevent VF recurrences but their effect on long-term clinical outcomes remains uncertain. Previously, amiodarone has been suggested to improve short-term survival in patients with OHCA and refractory VF as compared with placebo (5) and lidocaine (6). The randomized controlled Amiodarone, Lidocaine or Neither for Out-Of-Hospital Cardiac Arrest Due to Ventricular Fibrillation or Ventricular Tachycardia (ROC ALPS) trial found that neither amiodarone nor lidocaine resulted in a significantly higher rate of survival to hospital discharge or favorable neurologic outcome than the rate with placebo among patients with shockable OHCA (7). Although ROC ALPS was a rigorous pragmatic randomized trial, its highly structured protocol, specific enrollment criteria, and conduct within selected emergency medical services (EMS) systems may limit generalizability to the broader population of patients receiving routine prehospital care (8). Results from recent retrospective analyses suggest potential treatment benefits with lidocaine over amiodarone in patients with both in-hospital cardiac arrest (IHCA) (9) and OHCA, but these studies may be vulnerable to bias due to residual confounding (10). Because treatment decisions may evolve during resuscitation in response to the patient’s clinical course (e.g., elapsed time since arrest), such time-dependent confounding is difficult to address with conventional analytic approaches. We therefore used a target trial emulation framework to approximate a randomized controlled trial structure and apply causal inference methods to reduce confounding, aiming to obtain more robust causal effect estimates from observational data.

Until now, there has been no universal agreement on the preferred antiarrhythmic drug in patients with shockable OHCA, which is reflected by conflicting treatment recommendations. The 2025 Advanced Life Support (ALS) guidelines from the European Resuscitation Council recommend amiodarone over lidocaine for the treatment of shockable OHCA (11), whereas guidelines from the 2025 American Heart Association state that amiodarone or lidocaine may be considered for VF that is unresponsive to defibrillation (12). Because recent retrospective studies contrasted the findings of randomized controlled trials and thereby questioned guideline recommendations favoring amiodarone, we performed a target trial emulation to investigate whether the use of lidocaine or amiodarone is associated with higher rates of survival to hospital discharge in shockable OHCA using real-world data contained in a large registry.

## METHODS

### Study Design and Setting

This target trial emulation was conducted using data from the Resuscitation Outcomes Consortium (ROC) Cardiac Epidemiologic Registry (Cardiac Epistry) Version 3 (from April 1, 2011, to June 30, 2015). The ROC Cardiac Epistry is a prospective, population-based registry that enrolled individuals with nontraumatic OHCA across eleven regions in North America. These included eight sites in the United States—Birmingham, Alabama; Dallas, Texas; Iowa City, Iowa; Milwaukee, Wisconsin; Pittsburgh, Pennsylvania; Portland, Oregon; San Diego, California; and Seattle/King County, Washington—and three sites in Canada—Ottawa and Toronto, Ontario, and Vancouver, British Columbia (13). The ROC Cardiac Epistry received approval from the Institutional Review Board at each participating site. For additional information, please see the **Supplemental Digital Content** (https://links.lww.com/CCM/I21). Data for this analysis were obtained from the National Heart, Lung, and Blood Institute Biologic Specimen and Data Repository Information Coordinating Center (BioLINCC). The present analysis was approved by the National Heart, Lung, and Blood Institute (BioLINCC Identification Number 15144). Upon consultation with the local ethics committee before project initiation, it was confirmed that formal ethical approval or a committee vote is not required for analyses of anonymized datasets from the National Heart, Lung, and Blood Institute Biologic Specimen and Data Repository. This study was conducted according to the Good Scientific Practice guidelines and principles set forth in the Helsinki Declaration of 1975.

The present study was conducted as a target trial emulation. Common observational studies are inherently susceptible to confounding, regardless of the study design, which limits their causal inference (14). A target trial framework involves the design of a hypothetical randomized trial, which is then emulated using observational data. When implemented appropriately, the target trial framework is expected to mitigate biases resulting from the incorrect application of observational data. Target trial emulations may be used to estimate the causal effect of interventions from observational data (15). This analysis was conducted in accordance with the Transparent Reporting of Observational Studies Emulating a Target Trial (TARGET) guideline (**Supplementary Tables 1** and **2**, https://links.lww.com/CCM/I21) (16).

### Patient Population

Patients from the ROC Cardiac Epistry Version 3 were screened for eligibility. This target trial emulation included patients 18 years old or older with nontraumatic, initially shockable OHCA, who received a total of three or more defibrillation attempts. Included patients were assigned to the lidocaine or amiodarone group based on the documented treatment received. Patients who received both drugs were excluded from this analysis. In clinical practice, a second antiarrhythmic agent may be introduced when the initial therapy fails to achieve an adequate response. An analysis based on the initially administered drug, analogous to an intention-to-treat approach, was not feasible because the available documentation did not reliably indicate which antiarrhythmic agent was administered first. Antiarrhythmic medication was given via IV or intraosseous access. Time zero was defined as first antiarrhythmic administration, which is typically given after the third defibrillation attempt.

To reflect current guidelines and clinical practice, this analysis included patients who received three or more defibrillation attempts, termed refractory VF. This approach mirrors the design of the ARREST (Amiodarone in the Out-of-Hospital Resuscitation of Refractory Sustained VentricularTachyarrhythmias) and ALIVE (Amiodarone vs. Lidocaine in Prehospital Ventricular Fibrillation Evaluation) trials but differs from ROC ALPS, in which patients were eligible for inclusion after receiving one or more shocks. **Supplementary Table 3** (https://links.lww.com/CCM/I21) compares the study design of this target trial emulation and the randomized controlled ROC ALPS trial. Participants from ROC ALPS, which was conducted during the time period of the Cardiac Epistry 3, were not included in this analysis. Patients were treated according to ALS guidelines from the American Heart Association at the time (2010), which preferred amiodarone over lidocaine as the first-line antiarrhythmic for refractory VF or pVT in OHCA (17). This may have influenced treatment selection and introduced confounding by indication. The target trial emulation approach, using inverse probability weighting, was applied to reduce this potential bias.

### Data Collection and Outcomes

The data collection methods for the ROC Cardiac Epistry have been described previously (13). All required data on episode-specific factors, patient demographics, clinical information, prehospital interventions and disposition, and patient outcomes were extracted from existing databases by trained personnel. Targeted reviews of EMS reports were conducted to improve the quality and completeness of the collected data.

The primary outcome of this target trial emulation was survival to hospital discharge. Secondary outcomes included arrival at the emergency department (ED), return of spontaneous circulation (ROSC) at ED arrival, and favorable neurologic outcome at discharge defined as a score on the modified Rankin scale (range, 0 [no symptoms] to 6 [death]) of 3 or less, indicating the ability to conduct daily activities independently or with minimal assistance (18).

### Statistical Analysis

Categorical variables are presented using numbers with percentage (%), continuous variables are summarized using means with sds or medians with interquartile ranges, depending on the data distribution.

To adjust for confounding, we used propensity scores to calculate inverse probability weights. To improve the stability of the inverse probability weighted estimates, stabilized weights were truncated at the 0.95 quantile, thereby limiting the influence of extreme weights from patients with a very low predicted probability of receiving the treatment they actually received. This approach reduces the impact of highly influential observations while preserving the overall study population. Propensity scores were calculated using logistic regression, with the choice of antiarrhythmic medication (lidocaine or amiodarone) being evaluated as the dependent variable and based on the following independent covariates: age in years, sex, race/ethnicity, location of cardiac arrest (public or nonpublic, and detailed localization), bystander witness status (witnessed or unwitnessed), resuscitation attempted by bystanders, automated external defibrillator shock delivered, elapsed time from call to ALS arrival, elapsed time from call to first EMS shock, use of a mechanical chest compression device, intraosseous access, prehospital administration of epinephrine, prehospital advanced airway placement, and elapsed time from call to first successful advanced airway. Covariates for the propensity score model were selected based on clinical and epidemiological relevance, with the aim of capturing key patient characteristics, arrest circumstances, time-dependent resuscitation factors, and prehospital interventions known to influence both antiarrhythmic drug selection and clinical outcomes in shockable OHCA. For each covariate, the standardized mean differences between groups were calculated before and after the inverse probability weighted adjustment. Standardized mean differences of less than 0.1 were interpreted as appropriate group balance (19). Probability differences with 95% CIs for all outcomes were calculated using a covariate-adjusted logistic regression model based on the inverse probability weighted data, yielding a doubly robust estimation approach that provides robustness to potential misspecification of either model (20). The covariates included in the multivariable logistic regression model were identical to those used to calculate the propensity score for inverse probability weighting.

Exploratory subgroup analyses were performed based on age (< 60 vs. ≥ 60 yr), bystander witness status (witnessed vs. unwitnessed), bystander cardiopulmonary resuscitation (CPR) status (bystander CPR vs. no bystander CPR), time to ALS arrival (< 8 vs. ≥ 8 min), and route of antiarrhythmic administration (IV vs. intraosseous). Route of administration was chosen due to potential differences in drug pharmacokinetics and resulting treatment efficacy. Two-sided *p* values of less than 0.05 were considered statistically significant. Due to the exploratory nature of this analysis, no adjustment of *p* values or CIs for multiple comparisons was made. Statistical analysis and visualization were performed using R (Version 4.1.2; R Foundation for Statistical Computing, Vienna, Austria, 2021) and RStudio (RStudio Team, Boston, MA, 2020).

## RESULTS

### Participants

Of 120,306 patients included in the ROC Cardiac Epistry 3, a total of 2,451 patients with shockable OHCA and three or more defibrillation attempts received either lidocaine (*n* = 987) or amiodarone (*n* = 1,464) as prehospital antiarrhythmic drug. The study flowchart is shown in **Figure 1**. Unadjusted and adjusted baseline characteristics are depicted in **Table 1**. Covariates were balanced between the two groups after inverse probability weighting. In the weighted pseudopopulation, patients in the lidocaine and amiodarone groups were comparable in mean age (64.1 ± 13.9 vs. 64.1 ± 14.6 yr), female sex (19.1% vs. 19.1%), public location of cardiac arrest (30.4% vs. 30.4%), bystander witness status (65.6% vs. 65.6%), attempted bystander CPR (53.3% vs. 53.4%), the frequency of intraosseous access (22.9% vs. 22.9%), and mean time from initial call to ALS arrival (8.9 ± 6.1 vs. 8.9 ± 5.3 min, respectively). **Supplementary Table 4** (https://links.lww.com/CCM/I21) shows the prehospital treatment in the two groups. Standardized mean differences of all covariates were below 0.1 in the adjusted analysis, suggesting an adequate group balance (Table 1 and **Supplementary Fig. 1**, https://links.lww.com/CCM/I21). **Supplementary Figures 2** and **3** (https://links.lww.com/CCM/I21) show the multicollinearity of covariates and the diagnostic plots of the regression model, respectively.

**TABLE 1.** Baseline Characteristics of Participants Before and After Weighting

Baseline Characteristic	Before Weighting			In Weighted Pseudopopulation^a^		
	Lidocaine (*n* = 987)	Amiodarone (*n* = 1464)	SMD	Lidocaine	Amiodarone	SMD
Age, yr, mean (sd)	63.3 (13.9)	64.8 (14.5)	0.104	64.1 (13.9)	64.1 (14.6)	0.002
Female sex (%)	21.3	17.1	0.107	19.1	19.1	0.001
Race and ethnicity (%)						
Hispanic	1.7	0.8	0.088	1.1	1.2	0.004
Asian	3.6	0.5	0.224	1.8	1.8	0.003
White	27.8	17.8	0.24	22.6	22.7	0.001
Black	5.7	4.3	0.063	5	5	0.002
Other race	0.1	0.1	0.01	0.1	0.1	0.001
Race unknown	61.9	77.1	0.335	69.7	70.1	0.008
Location (%)						
Nonpublic	70.7	68.2	0.054	69.3	69.3	< 0.001
Public	28.8	31.5	0.059	30.4	30.4	0.001
Unknown	0.5	0.3	0.037	0.3	0.3	0.009
Witnessed by bystander (%)						
Unwitnessed	25.5	25.4	0.003	25.7	25.7	< 0.001
Witnessed	66.1	66.1	0	65.6	65.6	< 0.001
Unknown	8.4	8.5	0.005	8.7	8.7	< 0.001
Resuscitated by bystander (%)						
Resuscitated by bystander	55.7	53.4	0.046	53.3	53.5	0.004
Not resuscitated by bystander	35.3	37.8	0.052	37.2	37.4	0.004
Resuscitation unknown	9	8.8	0.007	9.4	9	0.014
Bystander cardiopulmonary resuscitation attempted (%)	55.7	53.3	0.048	53.3	53.4	0.001
Time from initial call to, min, mean (sd)						
Advanced life support arrival time	8.6 (5.5)	9.4 (5.5)	0.154	8.9 (6.1)	8.9 (5.3)	< 0.001
First emergency medical services shock	11.5 (6.6)	11.6 (5.8)	0.002	11.1 (6.7)	11.1 (5.8)	0.001
Received automated external defibrillator shock (%)	4.6	3.9	0.033	4.3	4.3	0.002
Number of defibrillation attempts (%)						
3 shocks	21.3	16.4	0.125	19.7	17.8	0.051
4 shocks	20.7	16.4	0.11	20.6	17.6	0.078
5 shocks	14.9	16.2	0.036	14.4	16	0.045
≥ 6 shocks	43.2	51	0.158	45.2	48.7	0.069
Intraosseous access (%)	21.9	20.5	0.034	22.9	22.9	< 0.001

SMD = standardized mean difference.

a Based on inverse probability of treatment weighting using stabilized weights.

SMDs are presented as absolute values.

**Figure 1.**
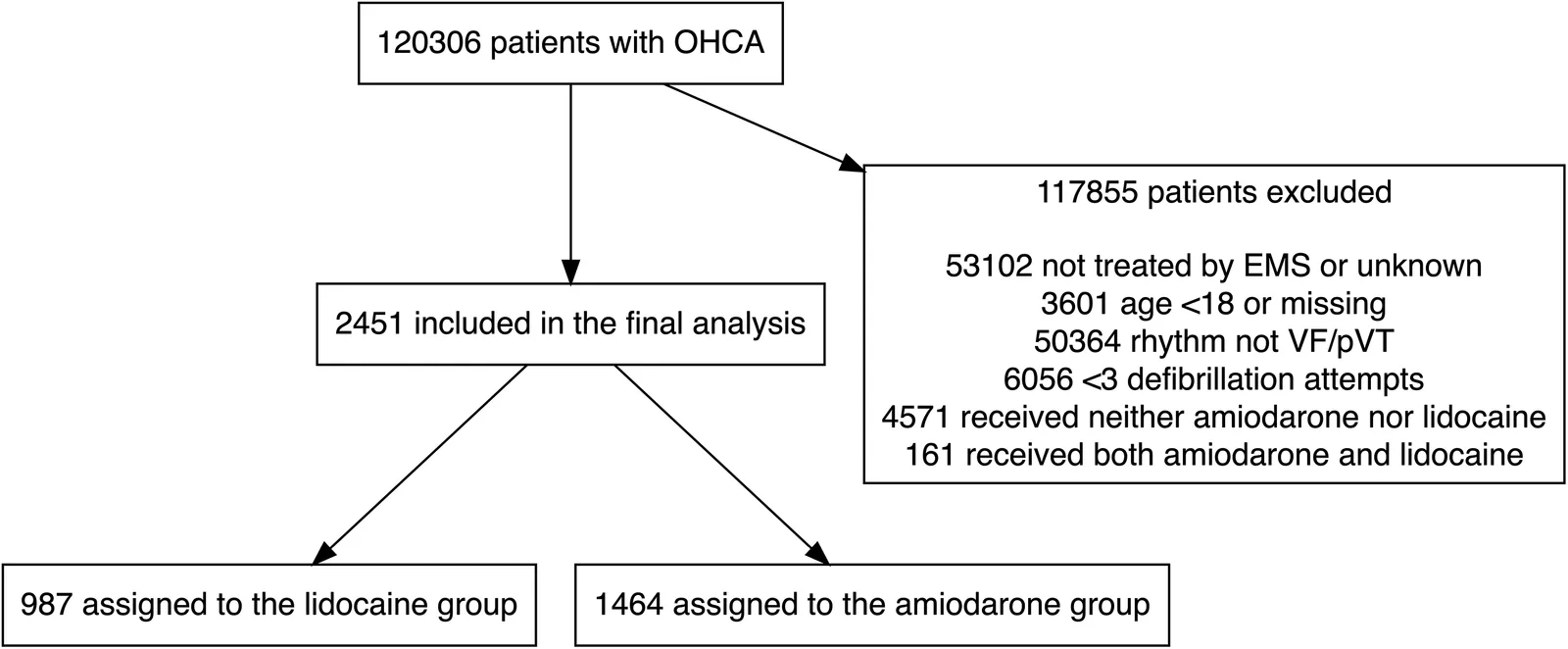
Flow chart of the study. EMS = emergency medical services, OHCA = out-of-hospital cardiac arrest, pVT = pulseless ventricular tachycardia, VF = ventricular fibrillation.

### Outcomes

In the adjusted analysis, the estimated probability of the primary outcome survival to hospital discharge was 26.2% (95% CI, 23.7–28.8%) in the lidocaine group (275/987) and 23.5% (95% CI, 21.3–25.6%) in the amiodarone group (329/1464), resulting in an adjusted percentage point difference of 2.8% (95% CI, –0.6% to 6.2%; *p* = 0.105; **Table 2**; **Fig. 2**).

**TABLE 2.** Primary and Secondary Outcomes

Outcome	Lidocaine (*n* = 987)		Amiodarone (*n* = 1464)		Group Comparison	
	*n*	Estimated Probability	*n*	Estimated Probability	Adjusted Probability Difference	*p*
Arrived at ED	834	83.63 (81.26–85.99)	1165	80.02 (77.96–82.08)	3.6 (0.45–6.76)	0.025
Return of spontaneous circulation at ED arrival	435	41.03 (38.03–44.03)	503	34.59 (32.16–37.03)	6.43 (2.55–10.32)	0.001
Survival to hospital discharge	275	26.24 (23.66–28.83)	329	23.46 (21.34–25.58)	2.78 (–0.58–6.15)	0.105
Favorable neurologic outcome at discharge (modified Rankin Scale ≤ 3)	182	17.68 (15.34–20.02)	233	16.18 (14.35–18.01)	1.5 (–1.47–4.47)	0.322

ED = emergency department.

All analyses are exploratory, and no adjustment for multiple comparisons was made.

**Figure 2.**
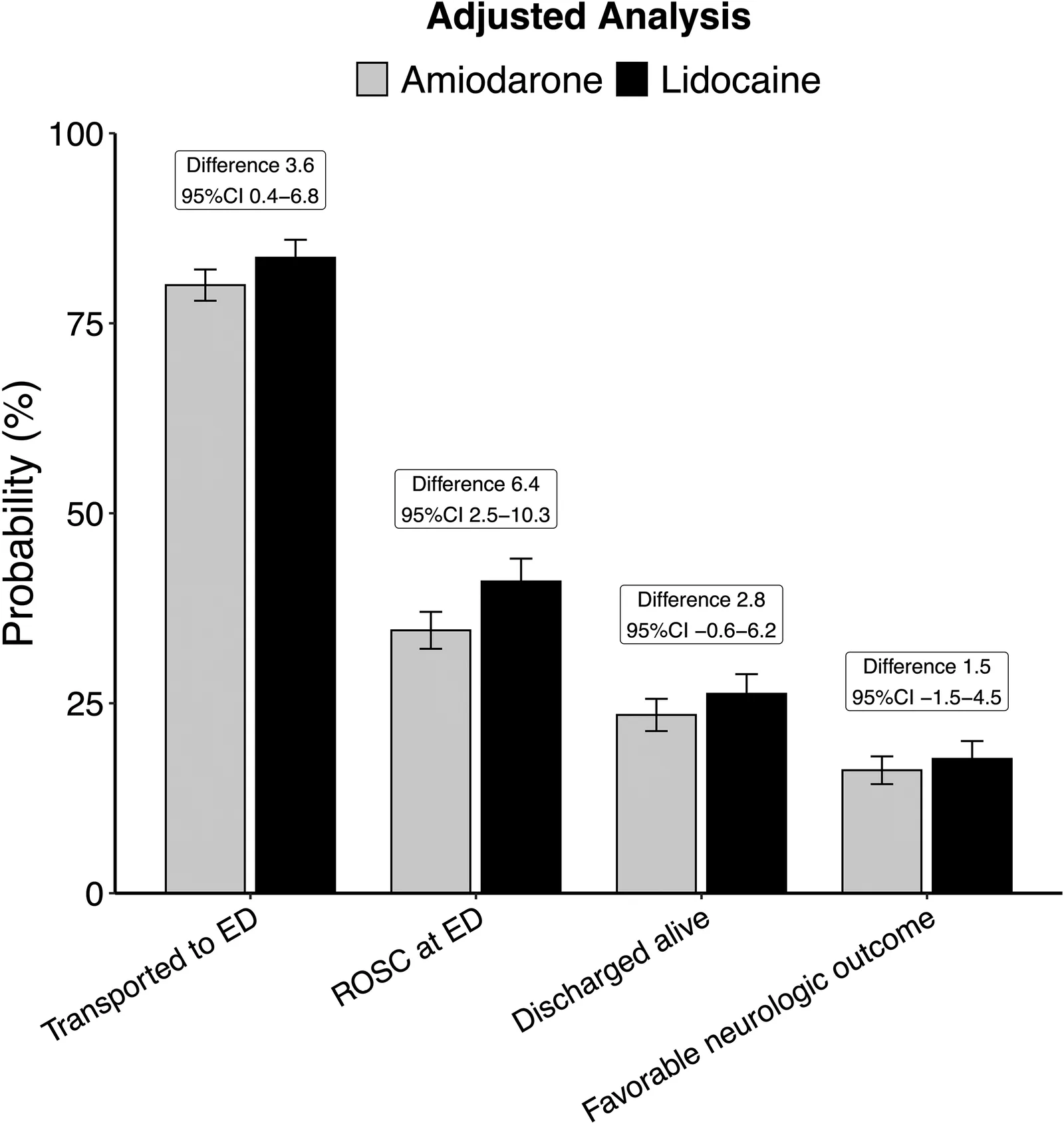
Outcomes of the adjusted analysis. All analyses are exploratory, and no adjustment for multiple comparisons was made. ED = emergency department, ROSC = return of spontaneous circulation.

The estimated probability of ED arrival (with or without ROSC) was greater in the lidocaine group (83.6%; 95% CI, 81.3–86.0%) than in the amiodarone group (80.0%; 95% CI, 78.0–82.1%; adjusted difference, 3.6%; 95% CI, 0.5–6.8%). The estimated probability of ROSC at ED arrival was greater in the lidocaine group (41.0%; 95% CI, 38.0–44.0%) than in the amiodarone group (34.6%; 95% CI, 32.2–37.0%; adjusted difference, 6.4%; 95% CI, 2.6–10.3%). No difference was observed in the estimated probability of favorable neurologic outcome at discharge between the lidocaine group (17.7%; 95% CI, 15.3–20.0%) and the amiodarone group (16.2%; 95% CI, 14.4–18.0%; adjusted difference, 1.5%; 95% CI, –1.5% to 4.5%). Unadjusted comparisons are presented in **Supplementary Table 5** and **Supplementary Figure 4** (https://links.lww.com/CCM/I21).

### Subgroup Analyses

**Figure 3** shows the subgroup analyses for the adjusted outcomes ROSC at ED arrival, survival to hospital discharge, and favorable neurologic outcome at discharge. The primary outcome survival to hospital discharge was similar across most subgroups, except for patients with witnessed cardiac arrest (adjusted difference, 5.3%; 95% CI, 1.1–9.5%), patients receiving bystander CPR (adjusted difference, 6.0%; 95% CI, 1.4–10.6%), and patients with intraosseous access (adjusted difference, 8.6%; 95% CI, 1.3–15.9%).

**Figure 3.**
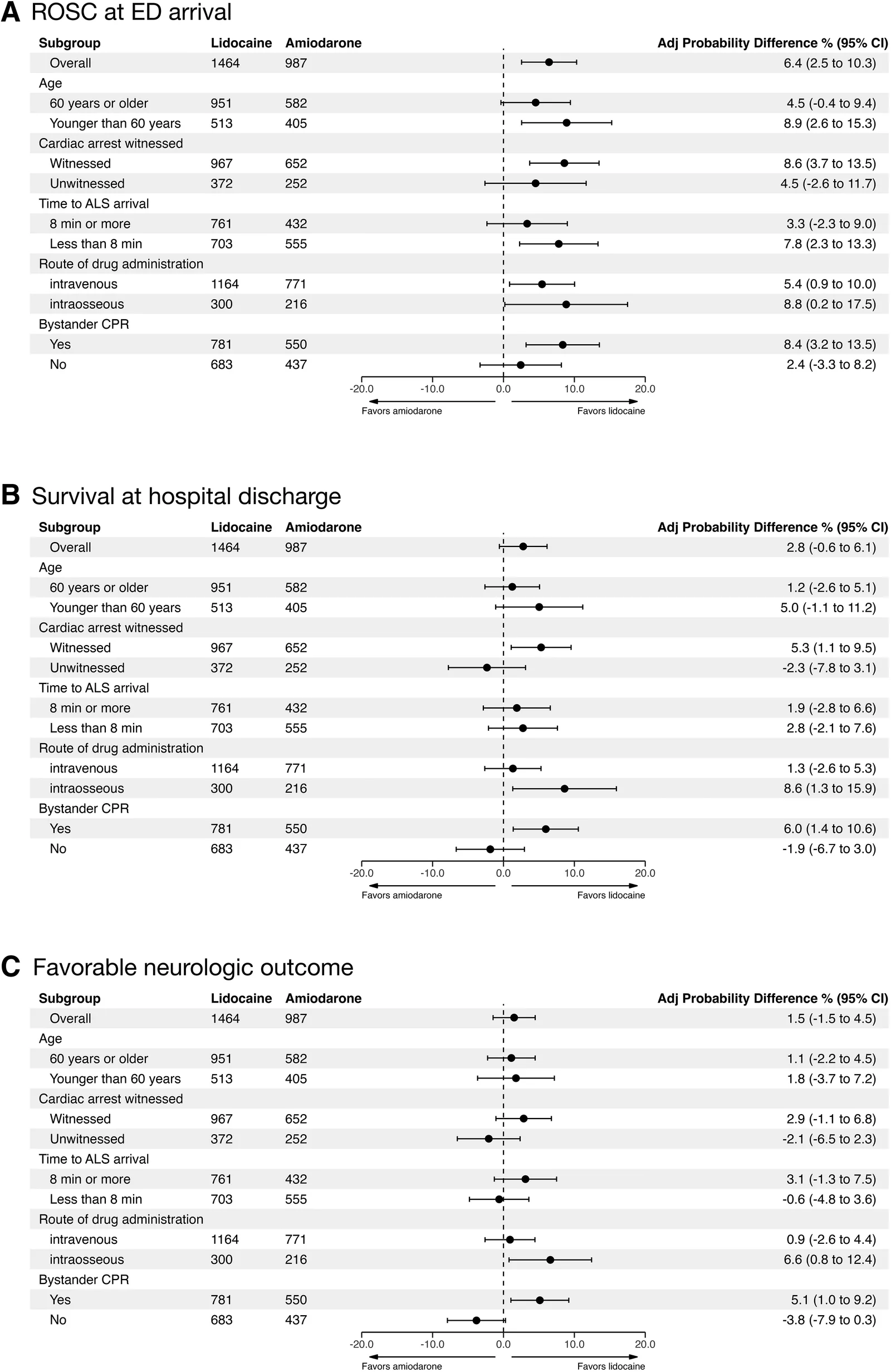
Subgroup analyses. Return of spontaneous circulation (ROSC) at emergency department (ED) arrival (**A**), survival to hospital discharge (**B**), and favorable neurologic outcome (**C**). All analyses are exploratory, and no adjustment for multiple comparisons was made. ALS = advanced life support, CPR = cardiopulmonary resuscitation.

## DISCUSSION

In this population-based target trial emulation of patients with shockable OHCA who received three or more defibrillation attempts, no statistically significant difference in survival or favorable neurologic outcome was observed between lidocaine and amiodarone; however, the wide CI for survival difference (95% CI, –0.6 to 6.2) cannot exclude a clinically meaningful benefit for lidocaine. Although patients who received lidocaine were more likely to be transported to the ED and achieve ROSC at ED arrival, this association did not translate into survival or favorable neurologic outcome at hospital discharge. In this target trial emulation, standardized mean differences were below 0.1 after inverse probability weighting and suggested adequate covariate balance between groups, indicating that the weighted pseudopopulation reasonably approximated a randomized comparison and supporting the interpretability of the adjusted estimates. This analysis supports the external validity and generalizability of the ROC ALPS findings in a large, real-world registry reflecting routine clinical practice variation.

Randomized, controlled, head-to-head comparisons of amiodarone vs. lidocaine for patients with OHCA and refractory VF were not definitive. In the 2002 ALIVE trial, survival to hospital discharge was low and did not differ significantly between treatment groups (5% in the amiodarone group vs. 3% in the lidocaine group) (6). Notably, the ALIVE trial had a limited sample size, also included patients with initial nonshockable cardiac rhythm (not restricted to shockable), and had a longer mean interval from dispatch to antiarrhythmic drug administration (25 min). In the 2016 ROC ALPS trial, survival to hospital discharge was similar between the amiodarone group and the lidocaine group but, similar to our study, patients who received lidocaine were more likely to achieve ROSC at ED arrival (39.9% vs. 35.9%; *p* = 0.07) (7). Two recent retrospective studies found lidocaine to be associated with a greater probability of ROSC in patients with IHCA (79.0% vs. 76.1%; *p* = 0.01) (9) and OHCA (36.0% vs. 30.4%; *p* < 0.01) (10), respectively, and further suggest a higher rate of survival to hospital discharge in patients who received lidocaine as compared with amiodarone.

Baseline characteristics were comparable between ALIVE, ROC ALPS, and the present study, with similar response times (time from initial call to ALS arrival was 7 min in ALIVE, 8 min in ROC ALPS, and 9 min in this study) and total defibrillation attempts (five in ROC ALPS, and five in this study). Although this study found no group difference in survival to hospital discharge and favorable neurologic outcome at discharge, the use of lidocaine was associated with a higher probability of ED arrival and ROSC at ED arrival as compared with amiodarone. These findings are consistent with results from ROC ALPS, which found a numerical increase of ROSC at ED arrival with lidocaine. While this difference did not translate into improved survival, achieving ROSC at ED arrival is considered an important prehospital resuscitation goal and has been associated with improved long-term outcomes (21, 22). The observed improvement in ROSC at ED arrival, but not in survival to hospital discharge, with lidocaine in both studies may indicate in-hospital treatment as a potential confounder. This may include post-resuscitation care quality, such as differences in targeted temperature management, hemodynamic support, or cardiac catheterization access/timing between groups or centers. Indeed, hospital care was not standardized and might have influenced outcomes. It is conceivable that a subset of patients who achieved ROSC with lidocaine had already sustained extensive hypoxic brain injury, precluding survival. This may help explain why higher rates of ROSC associated with lidocaine did not translate into corresponding improvements in survival to hospital discharge or favorable neurologic outcomes.

In exploratory subgroup analyses, lidocaine was associated with significant adjusted survival benefit among patients with witnessed cardiac arrest, receipt of bystander CPR, and those with intraosseous access. Patients with witnessed cardiac arrest or receipt of bystander CPR are populations generally characterized by earlier intervention and more favorable baseline physiology, in which a potential treatment benefit of lidocaine may be hypothesized. Intraosseous access might facilitate more reliable drug delivery, which could interact with the treatment effects of lidocaine and amiodarone. Because the outcomes of these subgroup analyses were not adjusted for multiple comparisons, they should be interpreted cautiously as hypothesis generating and require external validation due to the risk of false positives.

Our findings suggest that lidocaine and amiodarone are comparably effective for the treatment of VF unresponsive to defibrillation, which are consistent with evidence from randomized controlled trials (6, 7). Notably, our results contrast with recent retrospective studies that reported a potential advantage of lidocaine over amiodarone in patients with IHCA or OHCA (9, 10). In a Bayesian reanalysis of the ROC ALPS trial, both lidocaine and amiodarone demonstrated a high probability of improving survival and neurologic outcomes in patients with shockable OHCA, with a slight probability advantage for amiodarone (23).

We found that lidocaine was associated with a higher probability of ROSC at ED arrival, particularly when the time from the initial call to ALS arrival was under 8 minutes. However, this finding may be due to chance or unmeasured confounding, given previous studies suggesting that amiodarone is superior to lidocaine in achieving both ROSC at ED arrival and survival to hospital discharge when administered early (24, 25).

This study should be interpreted within the context of its limitations. First, although a target trial emulation framework was used, our study remains vulnerable to bias due to residual or unmeasured confounding, including factors that may have driven the choice between lidocaine and amiodarone during resuscitation. Further, institutional-level confounding, including local EMS drug availability or specific local protocols, cannot be excluded. We used inverse probability weighting to account for the potential for treatment indication bias, but it remains possible that treatment decisions were influenced by factors not measured in our study. Notably, all analyses were exploratory and not adjusted for multiple comparisons. Second, differentiation between defibrillation attempts occurring before and following antiarrhythmic drug administration was not possible. Third, baseline comorbidities of the included patients were unavailable. Although comorbidities are unlikely to influence the initial choice of antiarrhythmic treatment in refractory VF, they are strongly associated with the primary outcome of survival to hospital discharge. Patients with advanced cardiac disease, renal dysfunction, or poor functional status have lower survival probabilities irrespective of whether lidocaine or amiodarone is administered. The unavailability of baseline comorbidities limits our ability to completely adjust the estimated treatment effect for the strong prognostic influence of these factors on the primary outcome. Fourth, we were unable to differentiate between recurrent and shock-refractory VF; it is unknown whether the efficacy of the respective antiarrhythmic drug may depend on the type of refractory rhythm. Furthermore, the precise rhythm history and prior antiarrhythmic drug use was unknown, which could influence the EMS crew’s choice of antiarrhythmic agent.

## CONCLUSIONS

In this target trial emulation, no statistically significant difference in survival or favorable neurologic outcome was observed between lidocaine and amiodarone; however, the wide CIs cannot exclude a clinically meaningful benefit for lidocaine. These findings are consistent with randomized controlled trials, but residual confounding cannot be excluded.

## Supplementary Material
