# Supplementary material for: Lidocaine Versus Amiodarone in Patients With Shockable Out-of-Hospital Cardiac Arrest: A Target Trial Emulation
Source: Crit Care Med. 2026 Jul 15;54(10):2579–89. doi: 10.1097/CCM.0000000000007265 (PMC13632812; doi:10.1097/CCM.0000000000007265)
Supplement: Supplementary file 1 [file ccm-54-2579-s001.pdf]

SUPPLEMENTARY APPENDIX to:

Lidocaine versus amiodarone in patients with shockable out-of-hospital cardiac arrest: a target trial emulation

Institutional Review Board Approval

Details regarding the Institutional Review Board approval for the participating sites are provided in a research article on the rationale, development and implementation of the Resuscitation Outcomes Consortium (ROC) Cardiac Epidemiologic Registry (Cardiac Epistry).[1]

The ROC Cardiac Epistry is based on existing data sources (i.e., EMS and hospital records) and met requirements for minimal risk research in the United States and Canada. The registry protocol was reviewed and approved by 74 US Institutional Review Boards and 34 Canadian Research Ethics Boards as well as 26 EMS Services Institutional Review Boards. Additionally, approval in the form of a memorandum of understanding for data sharing was obtained from 24 hospitals and from 94 EMS services. The ROC data safety and monitoring board (DSMB) conducted semi-annual reviews.

Reference

1. Morrison LJ, Nichol G, Rea TD, et al. Rationale, development and implementation of the Resuscitation Outcomes Consortium Epistry-Cardiac Arrest. Resuscitation 2008;78(2):161-9. DOI: 10.1016/j.resuscitation.2008.02.020.

Supplementary Tables

Supplementary Table 1 Protocol of the index trial, specification of the target trial and its observational emulation (based on the TARGET guideline)

	Index trial	Target trial	Observational emulation
**Eligibility criteria**	*(i) 18 years of old or greater;<br>(ii) out-of-hospital cardiac arrest;<br>(iii) shock-refractory VF/VT, defined as confirmed persistent (non-terminating) or recurrent (restarting after successful termination) VF/VT after ≥1 shock anytime during resuscitation (inclusive of rhythms interpreted as shockable by an automated external defibrillator);<br>(iv) intravenous or intraosseous vascular access;*	*(i) 18 years of old or greater;<br>(ii) out-of-hospital cardiac arrest;<br>(iii) initial shockable cardiac rhythm and three or more defibrillation attempts;<br>(iv) intravenous or intraosseous vascular access;*	*(i) 18 years of old or greater;<br>(ii) out-of-hospital cardiac arrest;<br>(iii) initial shockable cardiac rhythm and three or more defibrillation attempts;<br>(iv) intravenous or intraosseous vascular access;*
**Treatment strategies**	*Amiodarone, lidocaine during out-of-hospital cardiac arrest; no exclusion criteria for treatment duration; patient exclusion if both treatments were given*	*Amiodarone, lidocaine during out-of-hospital cardiac arrest; no exclusion criteria for treatment duration; patient exclusion if both treatments were given*	*Amiodarone or lidocaine during out-of-hospital cardiac arrest; no exclusion criteria for treatment duration; patient exclusion if both treatments were given*
**Assignment procedures**	*Participants will be randomly assigned to either treatment strategy at baseline.*	*Participants will be randomly assigned to either treatment strategy at baseline.*	*Patients were retrospectively assigned to either the lidocaine group or the amiodarone group.*
**Follow-up**	*Baseline (time zero): randomization<br>Follow-up: time from trial drug administration until hospital discharge*	*Baseline (time zero): randomization<br>Follow-up: time from trial drug administration until hospital discharge*	*Baseline (time zero): first antiarrhythmic (amiodarone or lidocaine) administration*

			Follow-up: prehospital disposition, status at ED arrival, status at hospital discharge
**Outcome(s)**	*Primary outcome:*<br>*survival to hospital discharge*<br><br>*Secondary outcome:*<br>*survival with favorable neurologic status at discharge, defined as a score on the modified Rankin scale (range, 0 [no symptoms] to 6 [death]) of 3 or less*<br><br>*Mechanistic outcomes:*<br>*number of defibrillation shocks administered after receipt of the trial drug, return of spontaneous circulation at hospital arrival, hospital admission, hospital treatments, and time to withdrawal of life-sustaining treatments.*	*Primary outcome:*<br>*survival to hospital discharge*<br><br>*Secondary outcome:*<br>*survival with favorable neurologic status at discharge, defined as a score on the modified Rankin scale (range, 0 [no symptoms] to 6 [death]) of 3 or less*<br><br>*Mechanistic outcomes:*<br>*number of defibrillation shocks administered after receipt of the trial drug, return of spontaneous circulation at hospital arrival, hospital admission and hospital treatments.*	*Primary outcome:*<br>*survival to hospital discharge*<br><br>*Secondary outcomes:*<br>*ED arrival, return of spontaneous circulation (ROSC) at ED arrival, favorable neurologic outcome at discharge (defined as a score on the modified Rankin scale (range, 0 [no symptoms] to 6 [death]) of 3 or less)*
**Causal contrast(s)**	*Intention-to-treat effect, per-protocol effect*	*Intention-to-treat effect, per-protocol effect*	*Observational analogue of per-protocol effect*
**Identifying assumptions**	*n/a*	*n/a*	*(1) Within levels of the adjustment variables, groups receiving each treatment strategy at each time (baseline) have the same counterfactual risk of the outcome (conditional exchangeability). We adjusted only for characteristics that were found before trial intervention (in this case, administration of lidocaine or amiodarone)*

			(2) For every combination of the adjustment variables, there is a nonzero (ie, positive) probability of receiving each treatment strategy at each time (baseline).<br>*(3) The treatment strategies are sufficiently well-defined.*
**Data analysis plan**	*Survival: use of the z-test for comparison of binomial proportions with pooled variance*	*Survival: use of the z-test for comparison of binomial proportions with pooled variance*	*Inverse probability weighting using propensity scores; Adjusted risk differences*

Index trial outlined in more detail in the trial publication [*Amiodarone, Lidocaine, or Placebo in Out-of-Hospital Cardiac Arrest, Kudenchuk et al, NEJM 2016*]

Supplementary Table 2 The TARGET (TrAnsparent ReportinG of observational studies Emulating a Target trial) Checklist

Item no.		Checklist item			Location reported	
**Abstract**						
1	a	Identify that the study attempts to emulate a target trial using observational data. State the study objectives and briefly summarize the specified target trial.			Page 2	
	b	Report the data sources used for emulation.			Page 2	
	c	Summarize key assumptions, statistical methods, findings and conclusions.			Pages 2,3	
**Introduction**						
2	Background	Describe the scientific background of the study and the gap in knowledge.			Pages 5,6	
3	Causal question	Summarize the causal question.			Pages 5,6	
4	Rationale	Describe the rationale for emulating a target trial with the available data. Cite randomized trials informing the design of the target trial if applicable.			Pages 5,6	
**Methods**						
5	Data sources	Cite the data sources contributing to the analyses and for each one describe the following: original purpose, type, the geographic locations, setting and time-period. If relevant, describe how the data were linked or pooled.			Page 7	
6	**Target trial specification**<br>Specify the components of the target trial protocol that would answer the causal question.		7	**Target trial emulation**<br>Describe how the components of the target trial protocol were emulated with the observational data, including how all variables were measured or ascertained.	Supplementary Table 1	Pages 8,9
	**Eligibility criteria**			**Eligibility criteria**	Supplementary Table 1	Page 8, Supplementary Table 1
	a	Describe the eligibility criteria.	a	Describe how the eligibility criteria were operationalized with the data.		

**Treatment strategies**		**Treatment strategies**		Supplementary Table 1	Page 8, Supplementary Table 1
b	Describe the treatment strategies that would be compared.	b	Describe how the treatment strategies were operationalized with the data.		
**Assignment procedures**		**Assignment procedures**		Supplementary Table 1	Page 8, Supplementary Table 1
c	Report that eligible individuals would be randomly assigned to treatment strategies and may be aware of their treatment allocation.	c	Describe how assignment to treatment strategies was operationalized with the data.		
**Follow-up**		**Follow-up**		Supplementary Table 1	Page 9, Supplementary Table 1
d	Clarify that follow-up would start at time of assignment to the treatment strategies. Specify when follow-up would end.	d	Clarify that follow-up starts at the time individuals were assigned to the treatment strategies. Describe how the end of follow-up was operationalized with the data.		
**Outcomes**		**Outcomes**		Supplementary Table 1	Page 9, Supplementary Table 1
e	Describe the outcomes.	e	Describe how the outcomes were operationalized with the data.		
**Causal contrasts**		**Causal contrasts**		Supplementary Table 1	Pages 10,11, Supplementary Table 1
f	Describe the causal contrasts of interest, including effect measures.	f	Describe how the causal contrasts were operationalized with the data, including effect measures.		
**Identifying assumptions**		**Identifying assumptions**		Supplementary Table 1	Pages 10,11, Supplementary Table 1
g	Describe assumptions that would be made to identify each causal estimand. Describe the variables, if any, related to these assumptions.	g.i	For each causal estimand, describe assumptions made to identify it, including assumptions regarding baseline confounding due to lack of randomization.		
		g.ii	Describe how the variables related to these assumptions were operationalized with the data		Pages 10,11
**Data analysis plan**		**Data analysis plan**		Supplementary Table 1	Pages 10,11
h	For each causal estimand, describe the data analysis procedures and any associated statistical	h.i	For each causal estimand, describe the data analysis procedures and any associated statistical modelling		

		modelling assumptions, including approaches for handling missing data.	assumptions, including approaches for handling missing data.		
		h.ii	For each causal estimand, describe any additional analyses conducted to assess the sensitivity of the results to the choice of operationalizations, assumptions and analysis.		Pages 10,11
**Results**					
**8**	Participant selection	Report numbers of individuals assessed for eligibility, eligible, and assigned to each treatment strategy. A flow diagram is strongly recommended.		Page 12, Figure 1	
**9**	Baseline data	Describe the distribution of characteristics of individuals at baseline, by treatment strategy.		Page 12, Table 1	
**10**	Follow-up	Summarize length of follow-up and describe reasons for end of follow-up for each treatment strategy and causal contrast.		Pages 12,13	
**11**	Missing data	Describe the frequency of missing data in all variables, by treatment strategy when applicable.		Table 1	
**12**	Outcomes	Describe the frequency or distribution of each outcome, by treatment strategy.		Pages 12,13, Table 2, Figure 2	
**13**	Effect estimates	Report the effect estimates for each causal contrast with corresponding measures of precision, including both absolute and relative measures of effect, when applicable.		Table 2, Figure 2	
**14**	Additional analyses	Report results of all analyses to assess the sensitivity of the estimates to choices in operationalizations, assumptions and analysis.		Page 12, Figure 3	
**Discussion**					
**15**	Interpretation	Provide an interpretation of the key findings.		Page 14	
**16**	Limitations	Discuss the limitations of the study considering differences between the target trial and its emulation and the plausibility of assumptions, including assumptions regarding baseline confounding due to lack of randomization.		Pages 17,18	
**Other information**					
**17**	Ethics	Provide the institutional research board or ethics committee that approved the study and approval numbers, if relevant.		Page 7	

**18**	Registration	State whether, when and where the study protocol was registered.	Page 7
**19**	Sharing of study materials	Provide information on whether data, analytic code and/or other materials are accessible, and where and how they can be accessed.	Page 19
**20**	Funding sources	Provide the sources of funding and detail the role of the funders in the design, conduct and reporting of the study.	Page 19
**21**	Conflicts of interest	State any conflicts of interest and financial disclosures for all authors.	Page 19

Citation: Cashin AG, Hansford HJ, Hernán MA, Swanson SA, Lee H, Jones MD, et al. Transparent Reporting of Observational Studies Emulating a Target Trial: The TARGET Statement. JAMA. 2025; DOI: 10.1001/jama.2025.13350
© 2025 Cashin et al. This is an Open Access article distributed under the terms of the Creative Commons Attribution-NoDerivatives License (CC BY-ND 4.0), which permits redistribution, commercial and non-commercial, provided the work is passed along unchanged and in whole, with credit to the original author(s).

Supplementary Table 3 Comparison of the study design: the ROC ALPS trial and the target trial emulation

	**ROC-ALPS**	**Target trial emulation**
n	3026	2451
Treatment arms	Amiodarone (n=974)<br>Lidocaine (n=993)<br>Placebo (n=1059)	Amiodarone (n=1464)<br>Lidocaine (n=987)
Study design	Randomized, double-blind trial	Retrospective target trial emulation of prospectively collected data
Assignment procedures	1:1:1 randomization in permuted blocks of concealed size and stratified according to participating site and agency	Target trial emulation<br>Covariate-adjustment for confounding
Outcomes	Primary outcome:<br>survival to hospital discharge<br><br>Secondary outcome:<br>survival with favorable neurologic status at discharge, defined as a score on the modified Rankin scale (range, 0 [no symptoms] to 6 [death]) of 3 or less<br><br>Mechanistic outcomes:<br>number of defibrillation shocks administered after receipt of the trial drug, return of spontaneous circulation at hospital arrival, hospital admission, hospital treatments, and time to withdrawal of life- sustaining treatments.	Primary outcome:<br>survival to hospital discharge<br><br>Secondary outcomes:<br>ED arrival, return of spontaneous circulation (ROSC) at hospital arrival, favorable neurologic outcome at discharge
Follow-up	until hospital discharge	until hospital discharge
Inclusion criteria	(i) 18 years of old or greater;<br>(ii) out-of-hospital cardiac arrest;<br>(iii) shock-refractory VF/VT, defined as confirmed persistent (non-terminating) or recurrent (restarting after successful termination) VF/VT after ≥1 shock anytime during resuscitation	(i) 18 years of old or greater;<br>(ii) out-of-hospital cardiac arrest;<br>(iii) initial shockable cardiac rhythm and 3 or more defibrillation attempts<br>(iv) intravenous or intraosseous vascular access;

	(inclusive of rhythms interpreted as shockable by an automated external defibrillator);<br>(iv) intravenous or intraosseous vascular access;	
Exclusion criteria	(i) prior receipt of open-label amiodarone or lidocaine during resuscitation;<br>(ii) known hypersensitivity to amiodarone or lidocaine;<br>(iii) an advance directive;<br>(iv) member of protected population (child, pregnant, prisoner);<br>(v) study-provided bracelet denoting wish to be excluded from participation.	(i) receipt of both amiodarone and lidocaine
Additional analyses	Intention-to-treat analysis (primary analysis)<br>Per-protocol analysis<br>Subgroup analysis (witness status, receipt of bystander CPR, arrest location (public versus private), time-to-study drug, route of administration (intravenous versus intraosseous), treatment arm of the concurrent chest compression trial, site-specific baseline survival (survival rate in previous ROC PRIMED trial with a shockable initial rhythm and two or more EMS shocks), and the practice of antiarrhythmic drug administration (study drug given during ongoing CPR immediately following a second or later shock, versus study drug given after a brief pause in CPR to confirm the presence of VF/VT following 1 or more shocks)	Unadjusted versus adjusted estimates<br>Subgroup analysis age (59 years or younger versus 60 years or older), witness status, receipt of bystander CPR, arrest location (public versus private), time-to-arrival of ALS, route of administration (intravenous versus intraosseous)

Supplementary Table 4 Prehospital treatment

Variable	Overall	Lidocaine	Amiodarone
n	2451	987	1464
Endotracheal intubation (n [%])	1717 (70.1)	659 (66.8)	1058 (72.3)
Prehospital epinephrine given (n [%])	2335 (95.3)	929 (94.1)	1406 (96.0)
Total epinephrine dose, mg (median [IQR])	4.00 [3.00, 5.00]	4.00 [2.00, 5.00]	4.00 [3.00, 6.00]
Prehospital bicarbonate given (n [%])	551 (22.5)	255 (25.8)	296 (20.2)
Prehospital atropine given (n [%])	318 (13.0)	141 (14.3)	177 (12.1)
Prehospital pacing (n [%])	87 (3.5)	22 (2.2)	65 (4.4)
Number of EMS shocks (median [IQR])	5.00 [4.00, 7.00]	5.00 [4.00, 7.00]	6.00 [4.00, 8.00]
Total number of shocks (median [IQR])	5.00 [4.00, 7.00]	5.00 [4.00, 7.00]	6.00 [4.00, 8.00]
Total number of shocks (n [%])			
3 shocks	450 (18.4)	210 (21.3)	240 (16.4)
4 shocks	444 (18.1)	204 (20.7)	240 (16.4)
5 shocks	384 (15.7)	147 (14.9)	237 (16.2)
6 or more shocks	1173 (47.9)	426 (43.2)	747 (51.0)

Supplementary Table 5 Primary and secondary outcomes (unadjusted)

Outcome	Lidocaine (n=987)		Amiodarone (=1464)		Group Comparison	
	N	Estimated Probability	N	Estimated Probability	Adjusted Probability Difference	P Value
Arrived at ED	834	84.5 (82.24-86.76)	1165	79.58 (77.51-81.64)	4.92 (1.86-7.98)	0.002
ROSC at ED arrival	435	44.07 (40.98-47.17)	503	34.36 (31.93-36.79)	9.72 (5.78-13.65)	<0.001
Survival to hospital discharge	275	27.86 (25.07-30.66)	329	22.47 (20.33-24.61)	5.39 (1.87-8.91)	0.003
Favorable neurologic outcome at discharge (mRS≤3)	182	18.44 (16.02-20.86)	233	15.92 (14.04-17.79)	2.52 (-0.54-5.58)	0.106

Note: All analyses are exploratory, and no adjustment for multiple comparisons was made.

Supplementary Figures

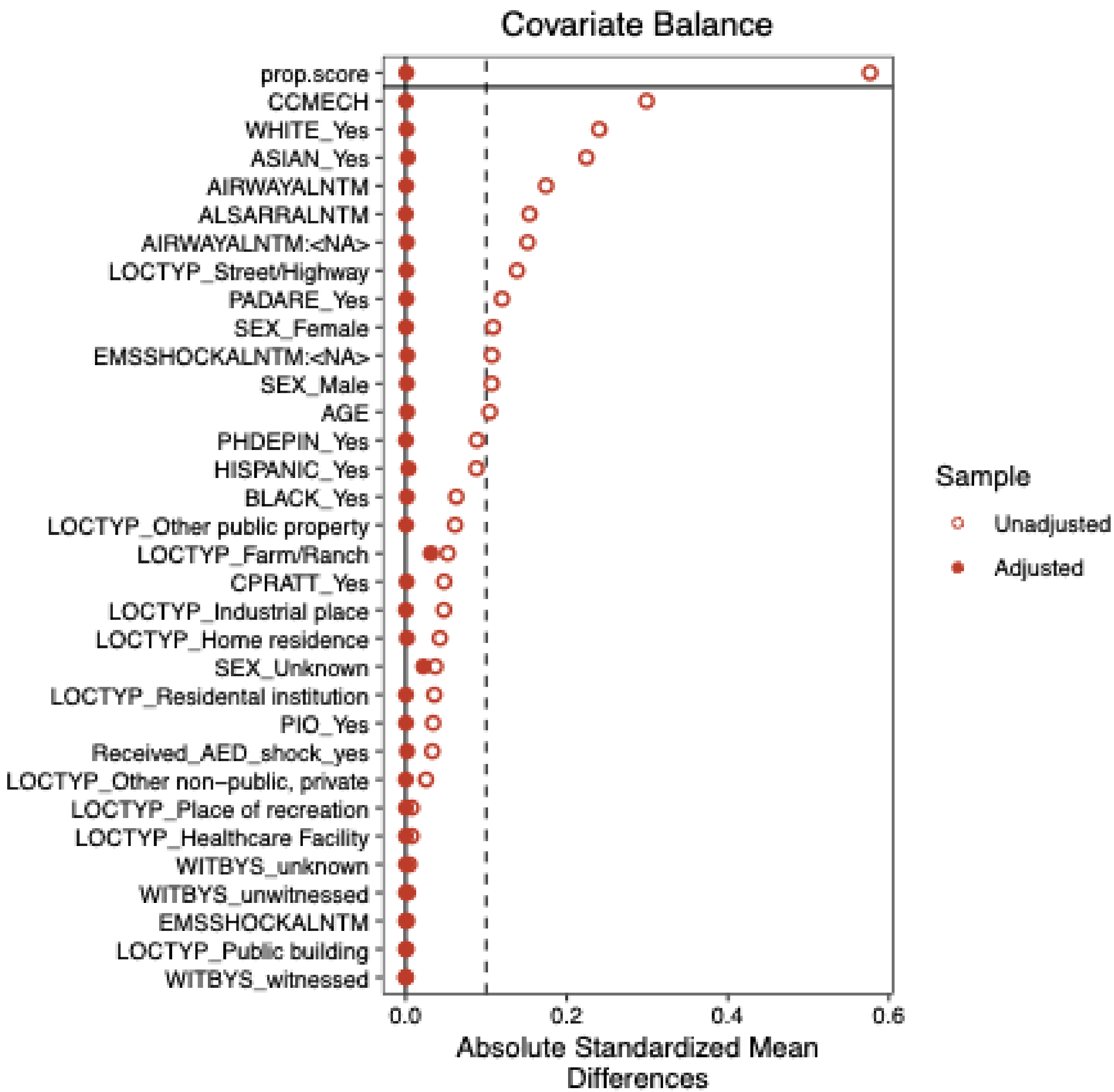

Supplementary Figure 1 Assessment of covariate balance before (empty circles) and after (full circles) inverse probability weighting.

The vertical dashed lines indicate the usual threshold for acceptable covariate balance, which corresponds to an absolute standardized mean difference (SMD) of 0.1.

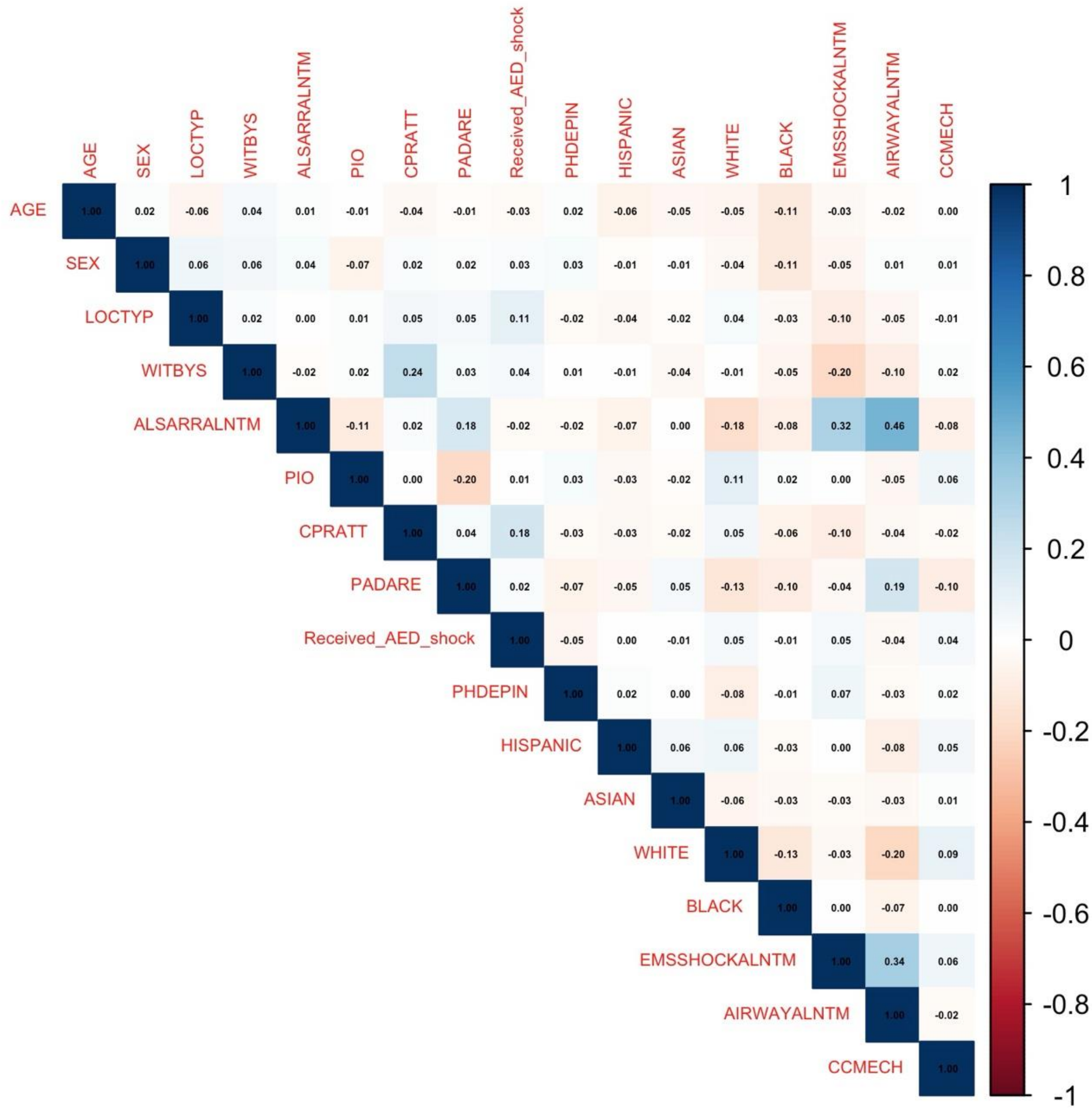

Supplementary Figure 2 Multicollinearity of covariates used in the weighting and the generalized linear model for the overall cohort

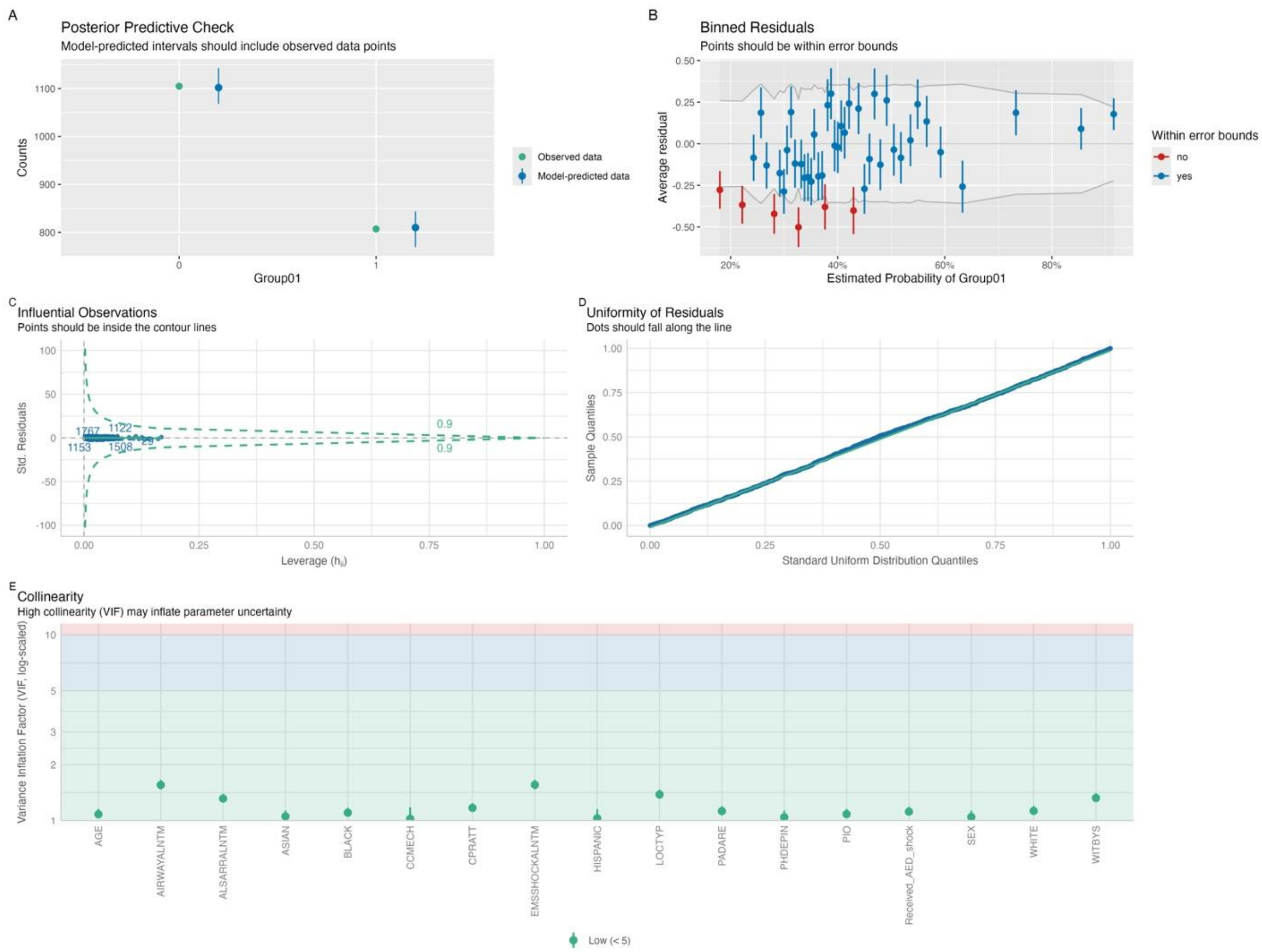

Supplementary Figure 3 Diagnostic plots of the generalized linear model used for the overall cohort

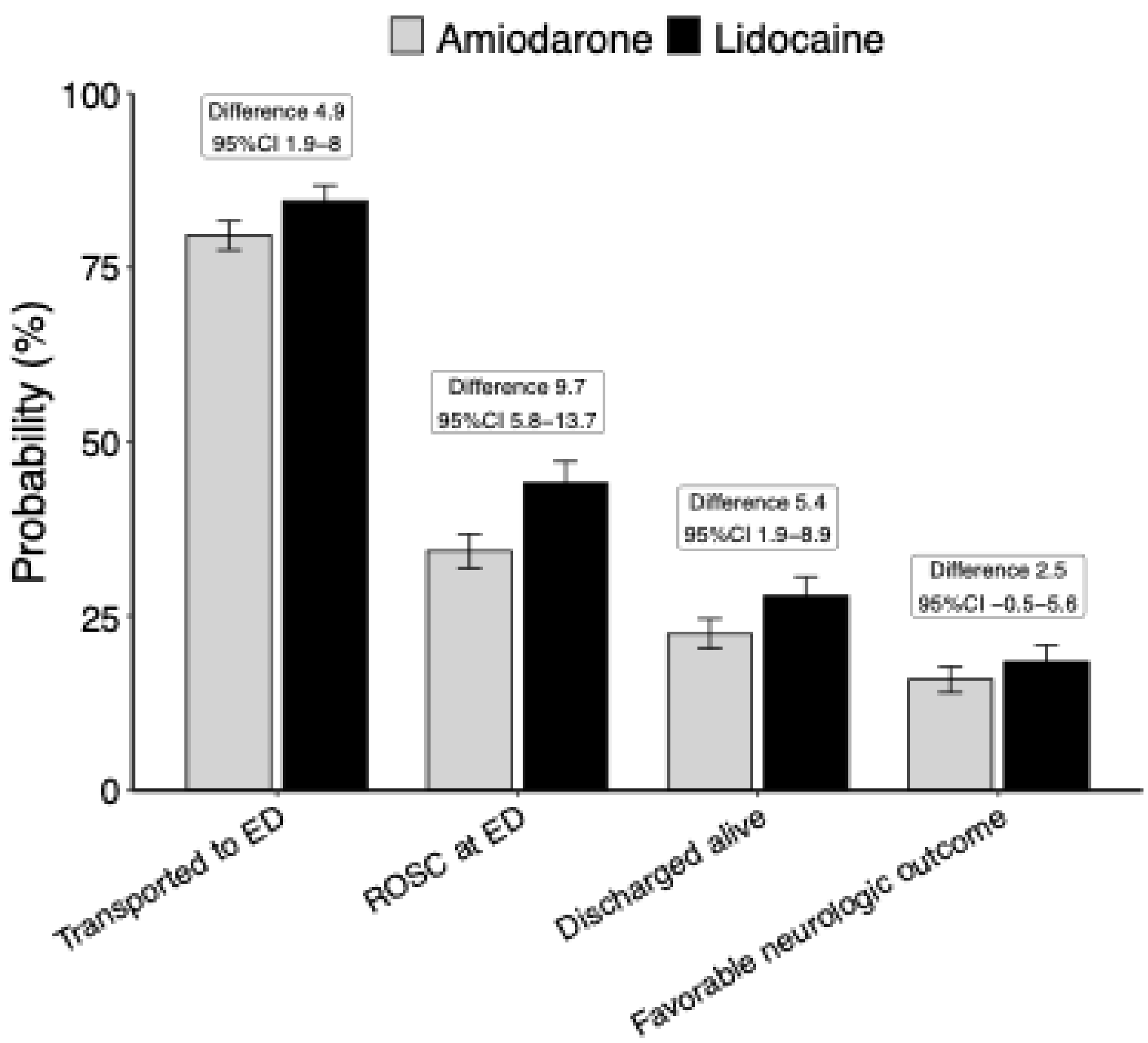

Supplementary Figure 4 Outcomes of the unadjusted analysis
